# Relationship of Interoceptive Accuracy with Craving, Personality Dimensions, and Alexithymia in Alcohol Use Disorder

**DOI:** 10.1192/j.eurpsy.2024.618

**Published:** 2024-08-27

**Authors:** E. Kahyacı Kılıç, M. B. Sönmez

**Affiliations:** ^1^ Edirne Sultan 1. Murat State Hospital; ^2^Trakya University School of Medicine, Edirne, Türkiye

## Abstract

**Introduction:**

Interoception encompasses processes that involve receiving, processing, and integrating bodily signals with external stimuli, ultimately influencing ongoing motivated behaviors. Disruptions in these interoceptive processes are believed to contribute to the development and progression of alcohol use disorder (AUD). Interoceptive accuracy (IAc), the objective dimension of interoception, has been shown to be decreased in patients with AUD. Traits linked to substance use vulnerability, such as personality dimensions and alexithymia, may be associated with decreased IAc.

**Objectives:**

Our objective was to compare the heartbeat perception (HBP) scores, as a measure of IAc, between abstinent inpatients with AUD and healthy controls. Additionally, we aimed to investigate potential associations between IAc and variables such as alcohol craving, personality dimensions, and alexithymia.

**Methods:**

The study comprised 48 abstinent inpatients with AUD and 68 healthy control subjects. All participants completed a heart rate tracking task, serving as an objective physiological measure of IAc. In addition to the IAc task, several assessments were administered to the patient group, including the Alcohol Use Disorders Identification Test (AUDIT), the Penn Alcohol Craving Scale (PACS), the Temperament and Character Inventory (TCI), and the Toronto Alexithymia Scale (TAS-20). Patients were recruited for a 28-day abstinence-based inpatient treatment program, and all assessments were conducted during the final week of hospitalization at the Alcohol and Substance Addiction Treatment Center in Trakya University School of Medicine (Edirne, Türkiye).

**Results:**

Patients’ HBP scores (mean ± standard deviation: 0.59±0.21) were significantly lower than those of healthy control subjects (0.74±0.15) (t=-4.469, p<0.001). The patients’ HBP scores showed significant negative correlations with AUDIT (r=-0.312, p=0.035), PACS (r=-0.361, p=0.019), and TAS-20 scores (r=-0.406, p=0.004). Additionally, there was a significant positive correlation between patients’ HBP scores and TCI self-directedness scores (r=0.371, p=0.009), and a near-significant correlation with TCI persistence scores (r=0.282, p=0.052). TCI novelty seeking, harm avoidance, reward dependence, cooperativeness, and self-transcendence scores did not significantly correlate with patients’ HBP scores (p>0.05).

**Conclusions:**

Our findings may support the hypothesis that interoceptive processes play a role in AUD, and that certain traits linked to vulnerability to alcohol use are associated with decreased IAc.

**Disclosure of Interest:**

None Declared

